# A Model for Migratory B Cell Oscillations from Receptor Down-Regulation Induced by External Chemokine Fields

**DOI:** 10.1007/s11538-012-9799-9

**Published:** 2013-01-08

**Authors:** Cliburn Chan, Matthew Billard, Samuel A. Ramirez, Harald Schmidl, Eric Monson, Thomas B. Kepler

**Affiliations:** 1 Department of Biostatistics and Bioinformatics, Duke University Medical Center, Durham, NC 27705 USA; 2 Thurston Arthritis Research Center, The University of North Carolina at Chapel Hill, Chapel Hill, NC 27599 USA; 3 Program in Computational Biology and Bioinformatics, Duke University, Durham, NC 27710 USA; 4 Duke University Visualization Technology Group, Duke University, Durham, NC 27708 USA; 5 Department of Microbiology, Boston University School of Medicine, 72E Concord St, Boston, MA 02118 USA

## Abstract

A long-standing paradigm in B cell immunology is that effective somatic hypermutation and affinity maturation require cycling between the dark zone and light zone of the germinal center. The cyclic re-entry hypothesis was first proposed based on considerations of the efficiency of affinity maturation using an ordinary differential equations model for B cell population dynamics. More recently, two-photon microscopy studies of B cell motility within lymph nodes in situ have revealed the complex migration patterns of B lymphocytes both in the preactivation follicle and post-activation germinal center. There is strong evidence that chemokines secreted by stromal cells and the regulation of cognate G-protein coupled receptors by these chemokines are necessary for the observed spatial cell distributions. For example, the distribution of B cells within the light and dark zones of the germinal center appears to be determined by the reciprocal interaction between the level of the CXCR4 and CXCR5 receptors and the spatial distribution of their respective chemokines CXCL12 and CXCL13. Computer simulations of individual-based models have been used to study the complex biophysical and mechanistic processes at the individual cell level, but such simulations can be challenging to parameterize and analyze. In contrast, ordinary differential equations are more tractable, but traditional compartment model formalizations ignore the spatial chemokine distribution that drives B cell redistribution. Motivated by the desire to understand the motility patterns observed in an individual-based simulation of B cell migration in the lymph node, we propose and analyze the dynamics of an ordinary differential equation model incorporating explicit chemokine spatial distributions. While there is experimental evidence that B cell migration patterns in the germinal center are driven by extrinsically regulated differentiation programs, the model shows, perhaps surprisingly, that feedback from receptor down-regulation induced by external chemokine fields can give rise to spontaneous interzonal and intrazonal oscillations in the absence of any extrinsic regulation. While the extent to which such simple feedback mechanisms contributes to B cell migration patterns in the germinal center is unknown, the model provides an alternative hypothesis for how complex B cell migration patterns might arise from very simple mechanisms.

**Electronic Supplementary Material:**

The online version of this article (doi:10.1007/s11538-012-9799-9) contains supplementary material, which is available to authorized users.

## Introduction

The evolution of high-affinity specific antibodies by long-lived B cells is driven by a process known as affinity maturation that occurs in the germinal center of lymph nodes. In this process, the germinal center (GC) is partitioned into a dark zone (DZ), consisting largely of rapidly dividing B cells known as centroblasts, and a light zone (LZ), consisting largely of B cells known as centrocytes interacting with follicular dendritic cells (FDC). It is believed that somatic hypermutation which introduces random changes in the antibody nucleotide sequence occurs within centroblasts in the DZ, while centrocytes in the LZ interact with and compete for immune complexes bound to FDC (Allen et al. 2007a; Shlomchik and Weisel 2012). A long-held hypothesis of *cyclic re-entry* is that the periodic migration of B cells from the DZ to the LZ and vice versa is critical for the efficiency of affinity maturation (Kepler and Perelson 1993; Kepler et al. 1993; Meyer-Hermann et al. 2001). FDCs present antigen bound on Fc receptor-captured antibodies on their cell surface, and centrocytes compete for binding to these antigens. Centrocytes with high affinity B cell receptors are more likely to successfully bind antigen and receive survival signals, while centrocytes with low affinity receptors fail to bind and undergo apoptosis. Successful centrocytes may then reenter the DZ for proliferation and another iteration of selection, or exit the germinal center as memory B cells or long-lived plasma cells.

How B cells migrate in the lymph node is hence critical for understanding the generation of high affinity long-lived memory and plasma cells that are the basis of humoral immunity. Naive B cells are believed to be attracted to the preactivation follicle primarily by the chemokine CXCL13, although lipid ligands that bind to the EBI2 receptor and CCR7:CCL19/CCL21 receptor-ligand interactions also modulate the B cell spatial distribution in the follicle (Gatto et al. 2011). In the post-activation germinal center, the migration of B cells between the DZ and LZ is driven by the chemokines CXCL12 found mainly in the LZ and CXCL13 in the DZ. These chemokines are recognized by the G-protein coupled receptors (GPCR) CXCR4 and CXCR5, with CXCR4 binding to CXCL12 and CXCR5 binding to CXCL13 (Allen et al. 2004, 2007a).

Pioneering work by Sally Zigmond has described receptor internalization (and resulting loss of sensitivity to a chemokine gradient) as an important aspect of GPCR-mediated chemotaxis (Zigmond 1981; Zigmond et al. 1982). Estimated receptor levels are in the 10^4^ range (10,000–50,000) of receptors per cell (Zigmond 1981). Upon ligand binding, GPCRs signal to G-protein, become phosphorylated by GPCR kinase (effectively desensitizing the receptor by dissociating G-protein subunits), and internalize via one of two major pathways. One internalization pathway is fast and involves clathrin-mediated endocytosis. The other is slower and uses a lipid-raft/caveolae pathway. The clathrin pathway involves the recruitment of arrestin to the receptor, which can act as a scaffold for further signaling events. Receptors internalized in either pathway can potentially be recycled, or degraded. How chemokine receptors respond to the local chemokine field over time is hence likely to be a major regulatory mechanism for the migration behavior of B cells. Indeed, the literature describes alterations in chemokine receptor expression balance as the fundamental basis for directional migration within the lymph node and germinal center (Allen et al. 2004; Hardtke et al. 2005; Reif et al. 2002).

With the advent of two-photon microscopy, we can now observe individual B cell dynamics in situ within a developing germinal center (Allen et al. 2007b; Schwickert et al. 2007; Hauser et al. 2007b; Victora et al. 2010). However, two-photon microscopy is restricted to the visualization of relatively small regions and short time-spans. Computational modeling is therefore a valuable adjunct for inference beyond these short time and space scales, providing mechanistic insight into long range/long duration phenomena such as the relationship between B cell migration patterns and the efficacy of somatic hypermutation (Kleinstein 2002; Meyer-Hermann et al. 2009; Figge and Meyer-Hermann 2011). As traditional ordinary differential equation (ODE) models ignore the spatial inhomogeneity of the chemokine fields, computational simulations of individual-based models (IBM) may be more appropriate vehicles for understanding how emergent behavior arises from the interactions of single B cells with their environment and other cells (Figge 2005; Bogle and Dunbar 2009; Germain et al. 2011; Beltman et al. 2011).

The detailed biology of chemotaxis is complex, and existing models of chemotaxis are in general either mechanistic or phenomenological (Palsson and Othmer 2000; Hauser et al. 2007a; Figge et al. 2008). We use phenomenological models in this paper as our interest is in the feedback between receptors and an external chemokine field, and not so much in the detailed mechanism of chemotaxis. Phenomenological models are typically based on some variation of a persistent random walk biased in the direction of the chemokine gradient (Weiner 2002). To bridge between deterministic and stochastic motility models in continuous time, we use the classical Langevin process stochastic differential equation formalism for persistent random walks to model chemotaxis and reduce to a deterministic version by removing the Wiener noise component where appropriate. We also explicitly incorporate GPCR desensitization by an external chemokine field in the Langevin process model.

Chemokine receptors are regulated on multiple levels, and receptor dynamics can be complex (Lauffenburger and Linderman 1996). As an illustrative example (Beyer and Meyer-Hermann 2008) present a detailed formalism (comprising 6 differential equations with 13 free parameters) to model the dynamics of a single receptor type interacting with its chemokine. Implementing a model with that degree of complexity would focus attention on detail and detract from our intention to show that very simple mechanisms suffice to induce complex migratory behavior. We therefore chose to derive a new phenomenological model for the receptor incorporating just ligand binding, constitutive and binding-induced down-regulation, and de novo synthesis. The use of singular perturbation analysis leads to the formulation of a single equation to model the dynamics of each chemokine receptor.

Based on the considerations above, we propose a simple ODE model of individual B cells coupled to static chemokine fields. We used the model to investigate the range of dynamical behaviors exhibited in the presence of static chemokine field distributions representing the DZ and LZ of the germinal center. Our hypothesis was that study of a phenomenological model integrating spatial chemokine distributions, receptor regulation, and chemotaxis could provide a template for understanding the broad-stroke dynamics of B cells in the germinal center. This would complement the use of IBM simulations to fill in the fine details and reveal unexpected emergent behavior resulting from individual cell interactions. For our model, we chose to include just three components—a spatial distribution of chemokines in one dimension, a model for the regulation of chemokine receptors, and a chemotactic model for cell locomotion.

This manuscript describes the application of the spatially-driven ODE model to explore the migratory response of B cells to chemokines in the germinal center. We show that chemokine-induced receptor down-regulation and receptor-mediated chemotaxis in the presence of a simple fixed spatial distribution of the relevant chemokines is sufficient to induce complex migratory patterns, including intrazonal and interzonal oscillations.

## Model Definition and Analysis

### Static Chemokine Fields

The chemokine-driven ODE model is a deterministic nonlinear dynamical system in one spatial dimension, in which chemotaxis of a single cell is modulated by the levels of two chemokine receptors that are reciprocally regulated by the static 1D spatial distribution of their cognate chemokines.

#### Spatial Distribution of Chemokines

Chemokine fields are set up by the expression of chemokines by stromal cells in the germinal center with dynamics determined by diffusion, absorption and degradation, but in the steady state over short periods of time, we make the assumption that the chemokine field is static. We further simplify by assuming that each chemokine field has a Gaussian distribution, and only consider the dynamics along the axis that runs through both the follicle or germinal center centroids. Chemokines are modeled as functions of the cell displacement *x*—even though the field is static, cells with different displacements respond to the *local* chemokine field. This representation of chemokine fields as a function of cell displacement is flexible—it is possible to set up arbitrarily complex chemokine fields in this system if necessary to model in vivo measurements, for example, by using mixtures of Gaussians to represent multimodal fields.

##### Germinal Center Model

**Fig. 1:**
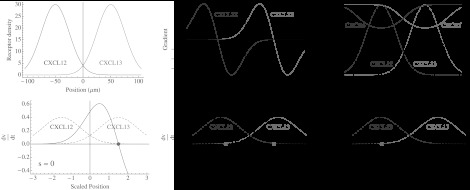
Characterization of static chemokine fields and dynamics of toy model. The *upper left panel* shows the static concentration of CXCL12 and CXCL13, and the *upper center panel* shows the gradient of CXC12 and CXC13. The *upper right panel* shows the steady state solutions for the receptor density induced by local chemokine concentration, with *dashed curves* representing chemokine concentrations (*blue* = CXCL12, *red* = CXCL13) and *solid curves* representing steady state receptor densities if a cell was kept fixed at that position (*blue* = CDCR4, *red* = CXCR5). The *lower panels* show the steady state solutions for the velocity *v* as *s*=*r*_1_/*r*_2_ varies. The *black curve* shows the rate of change of *v*, and the *red circles* indicate stable steady states. In the *left panel*, *s*=0 and the system only has a single steady state at *x*≈1.5. In the *middle panel*, *s*=1 and the system is bistable. Finally, in the *right panel*, *s*=4.5 and the system has lost the steady state at *x*≈1.5 and only the steady state at *x*≈−1.5 remains. Scaled chemokine concentrations represented by *f*_1_(*x*) and *f*_2_(*x*) are shown as *dashed lines* for reference (Color figure online)

The chemokine concentration is given as a function of the cell displacement *x*. For the examples in the paper, we use Gaussian distributions *f*_1_ and *f*_2_ to represent the CXCL12 and CXCL13 chemokine distributions respectively, i.e., 
1
2 where *c*_*i*_ and *w*_*i*_ determine the height and width of the distribution, and *k*=(*k*_1_+*k*_2_)/2 is the half-distance between the dark and light zones in μm. The chemokine concentrations and gradients for CXCL12 and CXCL13 are shown in the first two panels in the top row of Fig. 1.

### Toy Model for Receptor Regulation and Chemotaxis

We begin with a toy model for receptor regulation and chemotaxis to illustrate the basic requirements for chemokine-driven oscillations. We assume that the chemokine receptors are synthesized at a rate *π* and degraded at a rate *δ*. To couple the receptor dynamics to the chemokine field, we assume that receptors are also down-regulated at a rate proportional to the product of the receptor and the local cognate chemokine concentration. In other words, the chemokine drives the down-regulation of its receptor.

3 $$ \frac{dr_{i=1,2}}{dt} = \pi_{i} - r_{i} f_{i}(x) - \delta_{i} r_{i} $$

For chemotaxis, we assume that the cell velocity depends on the product of the receptor and the local cognate chemokine gradient, with a drag coefficient *γ*. 
4
5


### Analysis of Toy Model

In this section, we first analyze the toy model components individually to gain insight into the origin of specific dynamical behaviors, then integrate the components and explore the resulting system dynamics.

#### Regulation of Receptor Density

From Eq. (3), it follows that the steady state receptor density *r* ^SS^ at any given position *x* is given by

6 $$ r_i^{\text{SS}} = \frac{\pi_i}{\delta_i+f_i(x)} $$

In the rightmost upper panel of Fig. 1, we plot the steady state solution for the receptor density at a particular position. It is clear that the effect of binding-induced down-regulation is to decrease the receptor density the greatest where the chemokine concentration is highest. Where the level of cognate chemokine is low, synthesis of new receptor outpaces down-regulation, and the saturating density of receptor is achieved.

#### Stability of Cell Velocity

Next, we examine the dynamics of the velocity *v* with respect to relative changes in receptor concentration using a reduced undamped model (*γ*=0)

7 $$ \frac{dv}{dt} = s f_1'(x) + f_2'(x) $$

where we rescale so that *s*=*r*_1_/*r*_2_. We also assume that the chemokine fields *f*_1_(*x*) and *f*_2_(*x*) are standard normal distributions with centers set 1.5 units from the origin.

In the lower panels of Fig. 1, we plot the rate of change of the velocity as the position of a B cell is varied. There are three different sets of steady state solutions for *v* possible. When *s*, the ratio of the densities of the two chemokine receptors, is small (so that *r*_2_ dominates), there is a single steady state at the mean of *f*_2_(*x*). As *s* increases, a new steady state is created at the mean of *f*_1_(*x*) by a saddle-node bifurcation, and the system is bistable. As *s* continues to rise, the steady state at *f*_2_(*x*) vanishes in a reverse saddle node bifurcation, and the system becomes monostable again. This implies that under these conditions, the DZ, LZ or both can be equilibria for a B cell, depending only on the ratio of CXCR4 and CXCR5 expressed.

#### Coupling Receptor and Velocity Dynamics Results in Spontaneous Oscillations

Referring to the bottom panels of Fig. 1, we see how oscillations can arise from coupling of the receptor and velocity dynamics in the presence of opposing chemokine fields. Suppose a cell starts with a low density of CXCR4 and high CXCR5 at the CXCL12 peak. Under appropriate conditions, the only stable equilibrium is at the CXCL13 peak and the cell moves to the right (bottom left). When it reaches the CXCL13 peak, the chemokine drives the down-regulation of the CXCR5 receptor and CXCR4 is up-regulated. Now we are in the situation illustrated by the bottom right panel; the stable equilibrium at the CXCL13 peak vanishes, and the cell is forced to return to the CXCL12 peak, setting up a system where oscillations result.

#### Bifurcation Analysis

**Fig. 2:**
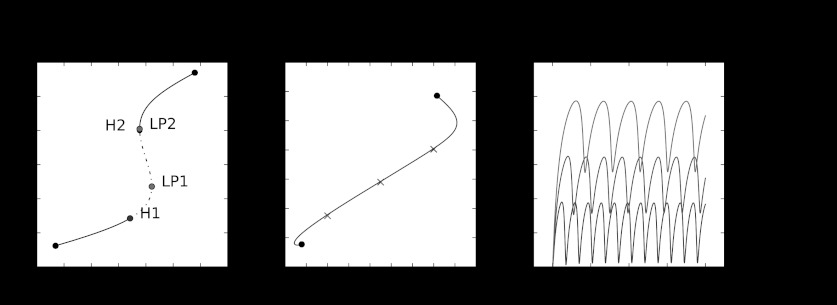
Bifurcation plots and sample trajectories for the toy model. *Left panel* shows the 1D bifurcation plot as the separation *k* between DZ and LZ is varied. Oscillations arise as *k* is increased above the threshold labeled as H1 where a supercritical Hopf bifurcation occurs. The *dotted line* segment indicates the parameter range for *k* where the equilibrium solution is unstable and oscillations exist. At approximately *k*=39.5, a subcritical Hopf bifurcation occurs at H2. The equilibrium becomes stable again at the saddle node bifurcation LP2 and there are no oscillations beyond this value. It is also possible to go beyond single parameters and identify parameter combinations where bifurcations occur as illustrated by the *middle panel* that shows the continuation of the Hopf bifurcation as *k* and the rate of synthesis of CXCR4 (*π*_1_) are simultaneously varied. The *right panel* shows sample trajectories for the pairs of (*k*, *π*_1_) parameters indicated by crosses in the *middle panel*, with trajectories for each parameter pair plotted matching the same *color cross*. Fixed parameters are chosen to be within reasonable biological ranges and to generate oscillations on the correct time scale (hours): *c*=20, *w*=15.7, *π*_2_=1, *δ*_1_=0.001, *δ*_2_=0.001, *γ*=1. In the *left panel*, *π*_1_=0.3, while in the *center panel*, the *red* (*k*=40, *π*_1_=0.35), *green* (*k*=45, *π*_1_=0.58) and *blue* (*k*=50, *π*_1_=0.805) *crosses* represent (*k*,*π*_1_) pairs that have oscillatory behaviors (Color figure online)

To better understand the conditions where the system exhibits oscillatory behavior, we can systematically study the dynamics under changes of parameters using software for continuation of equilibria (Dhooge et al. 2003; Clewley et al. 2007). Continuation software “follows” the equilibrium solution as some parameter is changed, and also checks for the occurrence of specific bifurcations at each parameter value. This allows us to identify parameter regions where interesting or desired system behavior is found, and provides insight into the parameter values where there is a qualitative change of behavior. Figure 2 shows the bifurcation plots from different parameter regions for the toy model.

### Biologically Motivated Phenomenological Model for Receptor Regulation and Chemotaxis

The toy model described above shows that a combination of receptor adaptation (modeled as down-regulation) and receptor-mediated chemotaxis can give rise to autonomous oscillations in the presence of a suitable static chemokine field. By design, the model abstracts away all other biological considerations. In this section, we describe simple biologically-motivated models that accommodate standard mass-action kinetics for receptor dynamics and chemotaxis with saturable chemokine receptor signals, and show that these models preserve similar autonomous oscillations.

#### Model for GPCR Regulation

**Fig. 3:**
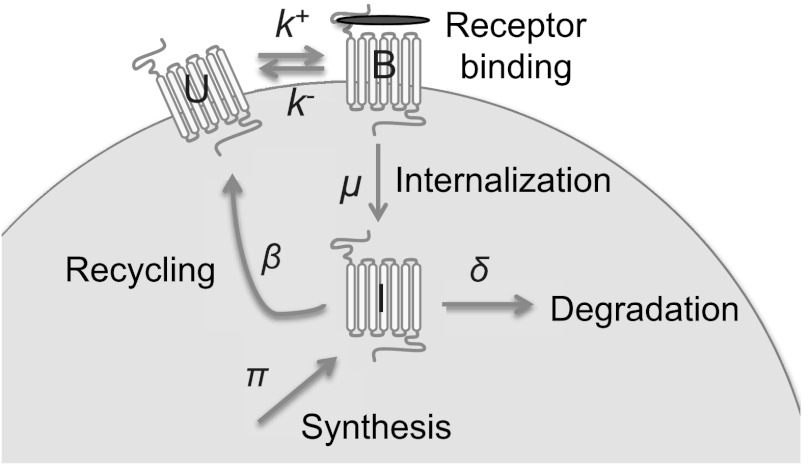
Schematic showing model for regulation of chemokine receptor levels on the cell surface. Unbound receptors (*U*) and bound receptors (*B*) levels are determined by association or dissociation with chemokine (shown as *red ellipse*) with rates *k* ^+^ and *k* ^−^, respectively. Bound receptors are endocytosed to become internalized receptors (*I*) with rate *μ* and internalized receptors can either be degraded with rate *δ* or recycled with rate *β*. Finally, new receptor synthesis occurs with rate *π* (Color figure online)

As discussed in a recent review by Bennett et al. (2011), the regulation of chemokine receptor levels is highly complex with multiple different processes that can affect GPCR levels and activity. However, the mechanism of migration is thought to be independent of transcription and regulated primarily by receptor trafficking dynamics in response to agonist binding (Schaeuble et al. 2012). Agonist-dependent desensitization in response to agonist binding results in GPCR endocytosis and degradation as discussed in the Introduction. Some of the internalized receptor may be recycled rather than degraded, and the path taken depends on both cell type and the duration of ligand engagement. While probably too slow a process to directly influence lymphocyte migration, new receptor synthesis is also essential for long-term maintenance of surface chemokine receptor levels. These processes of new receptor synthesis and agonist-induced internalization, recycling and degradation that together determine the dynamics of chemokine receptor expression are illustrated in Fig. 3. The mass-action kinetics corresponding to Fig. 3 are given by 
8
9
10 where the first-order degradation term *τ* for *U* is necessary to ensure that the unbound receptor remains finite in the absence of ligand. To simplify the model, we neglect the contribution of receptor recycling on the available intracellular pool. That is, we assume that *π*≫*μB*, and hence that *I* is constant. With these assumptions, we can derive the following model for the dynamics of the CXCR4 and CXCR5 receptors in the presence of their cognate chemokine ligands (full derivation given in Appendix A)

11 $$ \frac{dr_{i=1,2}}{dt} = \pi_{i} - \frac{r_{i} \tau_{i} \kappa_{i} f_{i}(x)}{1 + \kappa_{i} f_{i}(x)} - \delta_{i} r_{i} $$

where *κ* is a rescaled equilibrium association constant for GPCR:lignad interactions, and *δ*+*τ* is the removal rate for bound GPCR that incorporates the first-order degradation of unbound receptor *U*.

#### Chemotactic Model for Cell Locomotion

The model for B cell chemotaxis assumes that the cell velocity is governed by a chemotactic process biased by saturable chemokine receptor signals generated by receptor ligand interactions that depend on both ligand concentration and density. The velocity has a drag coefficient *γ*, and a tuning factor for the degree of responsiveness to the underlying chemokine field given by *χ*. The model equations (derived in Appendix B) are 
12
13


We have set the effective equilibrium association constant *ϵ* in the chemotactic model to be distinct from the value *κ* in the receptor regulation model to allow for differential coupling of bound receptor to signal transduction pathways involved in the two processes.

#### Bifurcation Analysis of Phenomenological Model

The full model is reproduced below for convenience. 
14
15
16
17


**Fig. 4:**
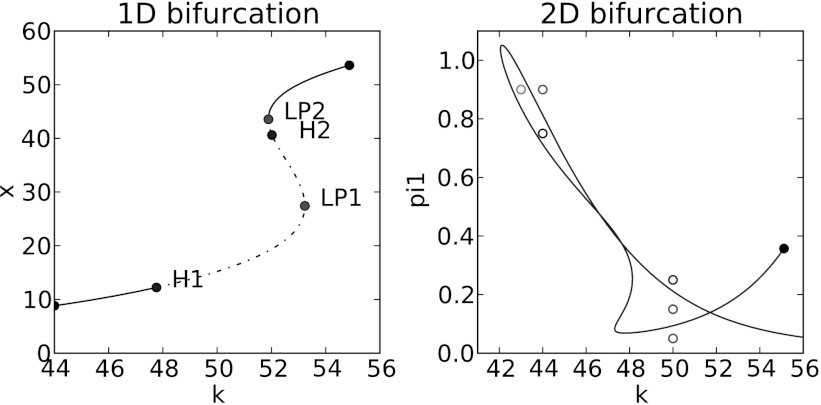
Bifurcation plots and sample trajectories for the reduced germinal center model. *Left panel* shows the 1D bifurcation plot as the separation *k* between DZ and LZ is varied. Oscillations arise as *k* is increased above the threshold labeled as H1 where a supercritical Hopf bifurcation occurs. The *dotted line segment* indicates the parameter range for *k* where the equilibrium solution is unstable and oscillations exist. At approximately *k*=52, a subcritical Hopf bifurcation occurs at H2. The equilibrium becomes stable again at the saddle node bifurcation LP2 and there are no oscillations beyond this value. It is also possible to go beyond single parameters and identify parameter combinations where bifurcations occur as illustrated by the *right panel* that shows the continuation of the Hopf bifurcation as *k* and the rate of synthesis of CXCR4 (*π*_1_) are simultaneously varied. Fixed parameters: *c*=10, *w*=25, *π*_2_=0.1, *τ*_1_=0.06, *τ*_2_=0.06, *δ*_1_=0.006, *δ*_2_=0.006, *κ*_1_=1, *κ*_2_=0.1, *ϵ*_1_=0.3, *ϵ*_2_=0.3, *χ*=28, *ζ*=1, *γ*=5. In the *left panel*, *π*_1_=0.15, while in the *right panel*, the *cyan* (*k*=43, *π*_1_=0.9), *black* (*k*=44, *π*_1_=0.75), and *green* (*k*=50, *π*_1_=0.15) *circles* represent (*k*, *π*_1_) pairs that have oscillatory behavior, while the *magenta* (*k*=44, *π*_1_=0.9), *blue* (*k*=50, *π*_1_=0.25), and *red* (*k*=50, *π*_1_=0.05) *circles* represent (*k*, *π*_1_) pairs that have steady state solutions. In particular, the *green circle* (*k*=50, *π*_1_=0.15) corresponds to a solution in the unstable equilibrium region of the *left panel*, where oscillatory behavior is predicted. Sample trajectories corresponding to these parameter pairs are shown in Fig. 7 (Color figure online)

As with the toy model, the bifurcation analysis suggests that spontaneous oscillations only occur for a rather restricted set of parameter combinations. For example, the leftmost panel of Fig. 4 shows that spontaneous oscillations only occur when the distance separating the simulated DZ and LZ fall within a narrow range.

#### Diversity of Dynamical Behaviors in 1D

**Fig. 5:**
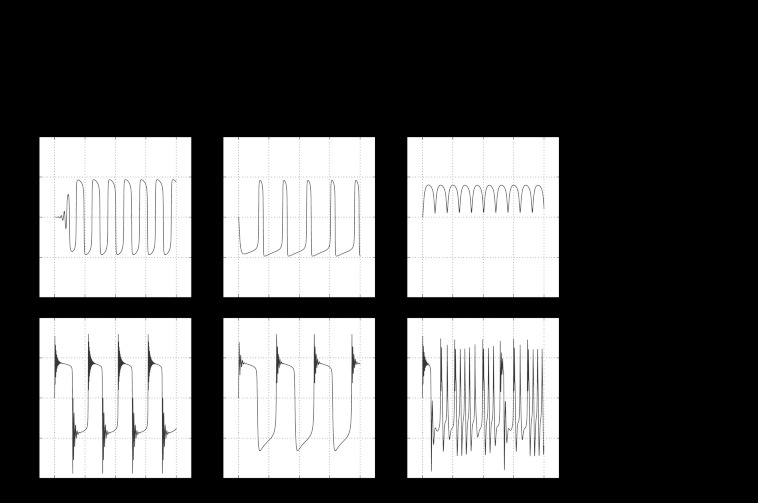
Diversity of oscillations with the phenomenological model. In each case, initial conditions were *x*=0,*v*=1,*r*_1_=1,*r*_2_=1. *Top row*: *Left panel* shows symmetrical oscillations, *center panel* shows asymmetrical oscillations and *right panel* shows oscillations confined to one zone. *Bottom row*: *Left panel* shows “nested” large and small oscillations in both zones, *center panel* shows “nested” small oscillations only in one zone, and *right panel* shows chaotic oscillations. Parameters: *Top left* (*c*=10, *w*=25, *π*_1_=0.15, *π*_2_=0.15, *τ*_1_=0.06, *τ*_2_=0.06, *δ*_1_=0.006, *δ*_2_=0.006, *κ*_1_=0.5, *κ*_2_=0.5, *ϵ*_1_=0.3, *ϵ*_2_=0.3, *χ*=28, *γ*=5, *k*=50); *top center* (*c*=10, *w*=25, *π*_1_=0.23, *π*_2_=0.15, *τ*_1_=0.06, *τ*_2_=0.06, *δ*_1_=0.006, *δ*_2_=0.006, *κ*_1_=0.5, *κ*_2_=0.5, *ϵ*_1_=0.3, *ϵ*_2_=0.3, *χ*=28, *γ*=5, *k*=50); *top right* (*c*=10, *w*=25, *π*_1_=0.15, *π*_2_=0.15, *τ*_1_=0.06, *τ*_2_=0.06, *δ*_1_=0.006, *δ*_2_=0.006, *κ*_1_=3.6, *κ*_2_=0.5, *ϵ*_1_=0.3, *ϵ*_2_=0.3, *χ*=28, *γ*=5, *k*=50); *bottom left* (*c*=5, *w*=22, *π*_1_=0.02, *π*_2_=0.004, *τ*_1_=0.005, *τ*_2_=0.01, *δ*_1_=0.001, *δ*_2_=0.001, *κ*_1_=10, *κ*_2_=10, *ϵ*_1_=3, *ϵ*_2_=0.2, *χ*=26.67, *γ*=0.03, *k*=45); *bottom center* (*c*=5, *w*=22, *π*_1_=0.05, *π*_2_=0.006, *τ*_1_=0.04, *τ*_2_=0.015, *δ*_1_=0.001, *δ*_2_=0.001, *κ*_1_=10, *κ*_2_=10, *ϵ*_1_=6, *ϵ*_2_=0.1, *χ*=3.33, *γ*=0.03, *k*=45); *bottom right* (*c*=5, *w*=22, *π*_1_=0.05, *π*_2_=0.0056, *τ*_1_=0.005, *τ*_2_=0.018, *δ*_1_=0.001, *δ*_2_=0.001, *κ*_1_=5.5, *κ*_2_=10, *ϵ*_1_=6, *ϵ*_2_=0.1, *χ*=16.17, *γ*=0.03, *k*=45)

A surprisingly rich variety of periodic behavior can be found in the 1D ODE model system. The range of behaviors include direct passage to a steady state equilibrium, damped oscillations to steady state and a variety of oscillatory behaviors with periods ranging from minutes to many hours. These oscillations may be from DZ to LZ, within a single zone or have components of both small intrazonal and large inter-zonal circulations. Oscillations can be highly asymmetrical, with a disproportionate amount of time spent in a single zone. Oscillations may even be apparently chaotic. Representative examples of the numerical simulations of displacement over time illustrating the diversity of oscillations are shown in Fig. 5.

### Individual-Based Model Simulations of Receptor Dynamics and Chemotaxis

Finally, we implemented the phenomenological model in a 3D IBM simulation of immune cells (Kepler and Chan 2007; Mitha et al. 2008), extending the chemotactic model to incorporate stochastic deviations. We show that very similar dynamical behavior is observed in the 3D simulation as in the simpler ODE models.

#### Stochastic Model for Chemotaxis

For the IBM, we rewrite the phenomenological model for chemotaxis as a stochastic differential equation in Ito form, giving rise to the following Langevin process equation 
18
19 where *dW* is the differential Wiener process.

There are three main differences between the phenomenological model and IBM simulation—the IBM is in 3D while the phenomenological model is 1D; cells in the IBM have a stochastic chemotactic motility model rather than a deterministic one (i.e., *σ*≠0); and there are extrinsic forces in the IBM when cells collide with each other or environmental boundaries. While we have closed form solutions for the spatial distribution of chemokines in the two models shown here, the simulation system uses a spatially discretized numerical approximation in order to generalize to arbitrary (and potentially evolving) chemokine distributions. Numerically, the differences between the IBM simulation and 1D phenomenological models are the use of a three-dimensional grid to store chemokine concentrations and gradients (5 μm per side voxels with trilinear interpolation between voxel centroids) as compared with values given by the closed forms *f*_1_ and *f*_2_ in the ODE model. In addition, cells in the IBM can have more complex behaviors such as division, death, and activation and the possibility of collision-induced forces when multiple cells are simulated. In the IBM simulation, cells are also constrained to be within a specified volume.

#### Dynamical Behavior in Individual-Based Simulation

**Fig. 6:**
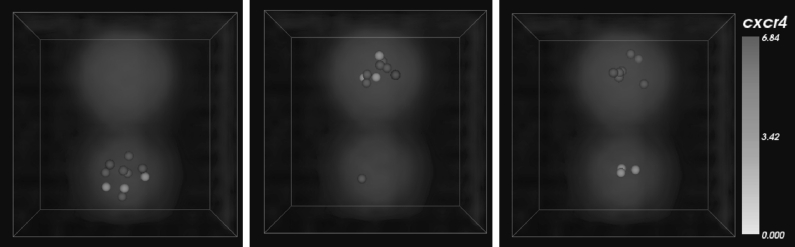
Snapshots of IBM GC B cell simulation. All B cells were started in the lower zone (DZ) and undergo spontaneous periodic motion between DZ and LZ as predicted by the phenomenological model. Chemokine concentrations in the DZ and LZ are volume-rendered, and the cell color shows density of CXCR4 receptors as indicated by the *color bar* (Color figure online)

Figure 6 shows three snapshots of the 3D IBM simulation. Oscillatory behavior is preserved in the presence of external forces and stochasticity, although of course, the periodicity is no longer synchronized between cells.

**Fig. 7:**
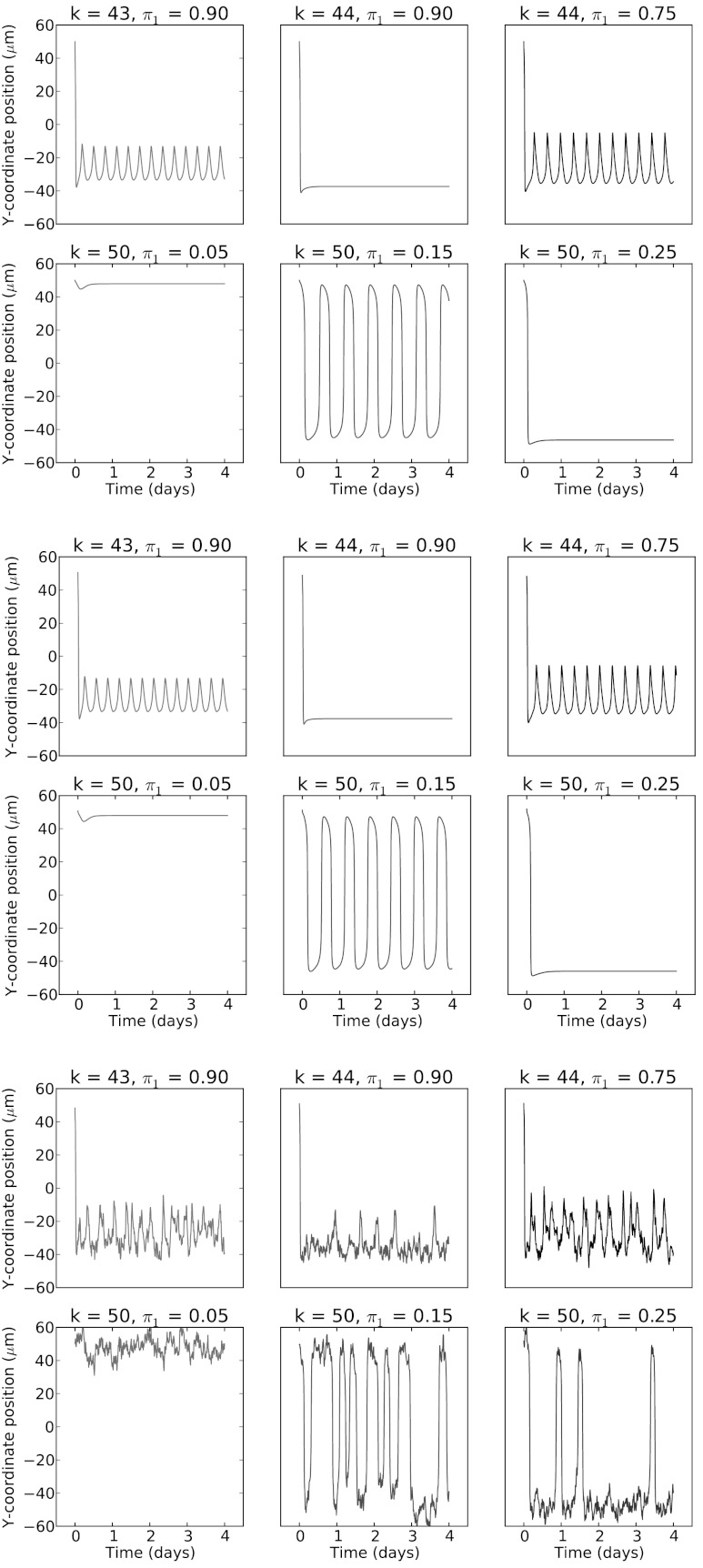
Comparison of sample oscillations from the 1D phenomenological model (*top*), IBM simulation with no stochasticity (*middle*) and IBM simulation with stochasticity (*bottom*). *Top*—Sample trajectories for the six (*k*, *π*_1_) parameter pairs shown in Fig. 4 found using a numerical ODE solver. *Middle*—Sample trajectories for the six (*k*, *π*_1_) parameter pairs shown in 4 obtained by running the 3D simulation for a single cell using identical parameter values ″ with *σ*=0. There is excellent agreement with the ODE solutions shown above. Very minor differences (e.g., slight change in periodicity) are likely due to spatial discretization (5 μm per side voxels with trilinear interpolation) and lower temporal resolution of the 3D simulation compared with the adaptive numerical integrator used in the ODE solution. *Bottom*—Sample trajectories for the six (*k*, *π*_1_) parameter pairs shown in Fig. 4 obtained by running the 3D simulation for a single cell again with identical parameters except for *σ*=2. The overall qualitative behavior is similar to that seen with *σ*=0, but the presence of stochasticity reveals the nearby attractor structure (see sporadic spikes in *top row*, *middle column*, and *bottom row*, *last column*) reminiscent of stochastic resonance

In the absence of stochasticity (Fig. 7 middle panel), the 3D simulation model behavior corresponds very closely to that of the 1D phenomenological model (Fig. 7 top panel). With stochasticity (Fig. 7 bottom panel), the 3D simulation behavior begins to diverge. However, the dynamical analysis of the minimal model remains informative for the 3D simulation behavior.

## Discussion

We have described a simple mathematical model of chemotaxis-driven B cell migration in the germinal center. The model incorporates a static chemokine field, chemokine-induced receptor modulation, and chemotaxis driven by the interaction of the chemokine receptor with the local chemokine concentration and gradient. The model is specified using coupled first-order differential equations, lending itself to detailed analysis using techniques from nonlinear dynamics. Using this basic setup, we investigated the dynamics of B cell migration under a simple chemokine field comprising of two Gaussian distributions representing CXCL12 in the light zone and CXCL13 in the dark zone of the germinal center.

In this simple germinal center model, we show that spontaneous oscillations between the light and dark zone can arise, and the periodicity can be tuned so that the residence times in the dark and light zones is consistent with experimental observations. An interesting prediction of the model is that for a fixed width of the chemokine fields, oscillations only occur for a narrow range of separations between the dark and light zone. When the light and dark zones are too close or far apart, no oscillations are observed. Oscillations can also be elicited in an alternative model where one receptor is fixed, and only one receptor is regulated by the chemokine field (supplementary Fig. A.1), but then the allowed range of separations is even narrower. This suggests that reciprocal regulation of both CXCR4 and CXCR5 receptors gives more robust oscillatory behavior than regulation of a single receptor.

While the simple mechanism of chemokine-driven receptor down-regulation is sufficient for inducing autonomous oscillations of some complexity, the extent to which such a mechanism contributes to B cell cycling in the germinal center is unknown. In fact, there is substantial evidence that B cell cycling in the germinal center is largely driven by extrinsic influences (e.g., B cell:FDC or B cell:T follicular helper cell interactions) that trigger differentiation programs regulating the expression of chemokine receptors. However, our model shows that surprisingly complex migratory patterns can emerge from very simple mechanisms, a recurring theme in the study of nonlinear dynamical systems. We believe that this provides a useful alternative perspective on the causal mechanisms of complex immune cell migration patterns, such as those observed in the germinal center.

This work was originally motivated by the desire to simplify IBM simulations of B cell behavior in order to gain insight into observed motility patterns and to facilitate parameter calibration. The 1D phenomenological model described in Eqs. (14)–(17) differ from the single cells in the 3D IBM simulations in the restriction to one dimension, the absence of a stochastic component, and the absence of collisions with other cells and the environment boundaries. However, we show that the phenomenological model effectively predicts the large-scale behavior of the IBM simulation when parameters are matched. Dynamical behaviors of interest can be rapidly identified in the phenomenological model configuration using bifurcation analysis and numerical simulations, and then studied in the more realistic 3D stochastic context with the IBM simulation using the same parameter values as the 1D phenomenological model. This is much more efficient than the brute-force search over parameter space otherwise necessary for IBM simulations, since such models are analytically intractable and highly demanding of computational resources. A caveat is that the extent to which such ODE-based model simplifications can replicate the dynamics of richer IBM that incorporate phenomena such as cell-cell interactions is not known. We conjecture that ODE models with mean-field approximations of cell-cell interactions will still be useful for providing insight into the parameters of these more challenging simulations and their calibration, and plan to investigate such approximations.

In conclusion, the ODE models for B cell motility described offer potential for a thorough analysis of the surprising complexity engendered by simple environment/cell interactions, and highlight the importance of considering the chemokine environment in understanding migration patterns of B cells. In addition, the ODE models provide flexibility to perform rapid prototyping of B cell migration dynamics, and may serve as a tractable bridge to more detailed IBM simulations.

### Electronic Supplementary Material

Below is the link to the electronic supplementary material.

Supplementary figures (PDF 166 kB)

## Appendix A: Model for GPCR Regulation

Let *r* be the density of unbound GPCR, and *r* ^∗^ the density of GPCR bound by its ligand, whose concentration is *l*. The dynamical equations for this system are

20

21

where *π* is the rate of production of GPCR where, *δ* is the removal rate for GPCR in the unbound state, and *δ*+*τ* is the removal rate for the bound GPCR.

$$\frac{\kappa}{\varepsilon} $$

is the forward rate constant for ligand binding,

$$\frac{1}{\varepsilon} $$

is the rate constant for dissociation. We use these expressions to facilitate taking the limit where the binding and dissociation reactions are much faster than the cellular processes. We reduce the complexity of the dynamical system by taking some of its subprocesses as occurring on a much faster time-scale than others. These fast subprocesses are treated as if in equilibrium with the more slowly varying components, thus eliminating dynamic degrees of freedom. The mathematics used is singular perturbation theory (Jones 1995). In the present case, the justification for making the approximation comes from the measured rates for the subprocesses. The binding and dissociation of CXCL12 and CXCR4 has been characterized using surface plasmon resonance, giving *k*_on_=4.20×10^5^ M s^1^, *k*_off_=8.24×10^−3^ s^1^, and *K*_*D*_=3.47×10^−8^ M, showing that the reactions equilibrate with a characteristic time of about 44 seconds for CXCL12 concentration equal to the reactions’ *K*_*d*_ (35 nM) (Vega et al. 2011). In contrast, the characteristic time for CXCR4 internalization upon binding by CXCL12 was found to be between 450 and 600 seconds (estimated from Signoret et al. 1997, Fig. 8A).

Define the total GPCR density *r*_*T*_≡*r*+*r* ^∗^ , whose rate equation is

22 $$ \frac{{d{r_T}}}{dt} = \pi - \delta{r_T} - \tau{r^*} $$

Equation (21) can now be written

23 $$ \frac{{d{r^*}}}{dt} = - ( {\delta + \tau} ){r^*} + \frac {\kappa}{\varepsilon} \bigl( {{r_T} - {r^*}} \bigr)l - \frac {1}{\varepsilon}{r^*} $$

We now perform the singular perturbation analysis, taking the limit *ε*→0. We first construct the “outer solution” by expanding the state variables as

24 $$ \begin{aligned}[c] {r_T} ( {t,\varepsilon} ) &\equiv{r_{T,0}} + \varepsilon {r_{T,1}} + O \bigl( {{\varepsilon^2}} \bigr) \hfill \\ {r^*} ( {t,\varepsilon} ) &\equiv r_0^* + \varepsilon r_1^* + O \bigl( {{\varepsilon^2}} \bigr) \end{aligned} $$

and then matching coefficients of *ε* in Eqs. (22) and (24). To lowest order, we have

25 $$ \begin{aligned}[c] \frac{{d{r_{T,0}}}}{dt} &= \pi - \delta{r_{T,0}} - \tau r_0^* \\ 0 &= \kappa \bigl( {{r_{T,0}} - r_0^*} \bigr)l - r_0^* \end{aligned} $$

the second of Eqs. (25) has solution

26 $$ r_0^* = \frac{{\kappa{r_{T,0}}l}}{{1 + \kappa l}} $$

so that the first of Eqs. (25) becomes

27 $$ \frac{{d{r_{T,0}}}}{dt} = \pi - \biggl( {\delta + \frac{{\tau\kappa l}}{{1 + \kappa l}}} \biggr){r_{T,0}} $$

To impose initial conditions, we must compute the “inner solution” obtained by rescaling time as

28 $$ T = \frac{t}{\varepsilon} $$

and letting

29 $$ {R_T} ( {T,\varepsilon} ) = {r_T} \bigl( {t(T, \varepsilon ),\varepsilon} \bigr) $$

etc.

Now, matching coefficients of *ε* in the resulting differential equations gives

30 $$ \begin{aligned}[c] \frac{{d{R_{T,0}}}}{dt} &= 0 \\ \frac{{dR_0^*}}{dt} &= \kappa \bigl( {{R_{T,0}} - R_0^*} \bigr)l - R_0^* \end{aligned} $$

Now the initial value problem in the inner solution has solution

31 $$ {R_{T,0}} ( T ) = {r_T} ( 0 ) $$

and

32 $$ R_0^* ( T ) = \biggl( {{r^*} ( 0 ) - \frac{{\kappa l{r_T}(0)}}{{1 + \kappa l}}} \biggr){e^{ - ( {1 + \kappa l} )T}} + \frac{{\kappa l{r_T}(0)}}{{1 + \kappa l}} $$

Matching the inner and outer solutions requires setting

33 $$ {r_{T,0}} ( 0 ) = \lim _{T \to\infty} {R_{T,0}} ( T ) = {r_{T,0}} ( 0 ) $$

and

34 $$ r_0^* ( 0 ) = \lim_{T \to\infty} R_0^* ( T ) = \frac{{\kappa l{r_T}(0)}}{{1 + \kappa l}} $$

Equation (11) from the text is recovered by dropping subscripts and substituting the chemokine fields for the ligand concentration,

35

Initial parameter values for the spatial measurements and receptor dynamics used for bifurcation analysis and numerical simulations were derived from the literature (Lin and Butcher 2008; Hauser et al. 2007a; Allen et al. 2004; Victora et al. 2010; Zigmond 1981; Hoffman et al. 1996; Ricart et al. 2011), or estimated when no experimental data was available.

## Appendix B: Model for Cellular Locomotion

The model for cellular locomotion starts with a third-order Langevin process in Ito form:

36

where *X*(*t*)∈ℝ^3^ is the cell’s position at time; *V*(*t*)∈ℝ^3^ and *P*(*t*)∈ℝ^3^ are the velocity and polarization, respectively. We use upper-case letters to remind us that these variables are stochastic. The effective drag coefficient is *γ*, and the polarization decorrelation rate is *ζ*. *Φ* is the external force exerted on the cell, and *Γ* is the signal due to an external orientation field. *dW* is a Wiener process generating fluctuations in the polarization, and *σ* controls the size of those fluctuations.

We proceed from here by providing a model for *Γ* in the case where the orientation field is due to an inhomogeneous chemokine distribution. We suppose that the binding of chemokine receptors on the cell’s surface generates a local signal, and the global orientation signal is the vector average of these signals over the cell’s surface.

37 $$ \varGamma\propto\int_{S}{{d^{2}}y \,{r^{*}} ( y )\widehat {n} ( y )} $$

where, as in Appendix A, *r* ^∗^ is the density of bound receptor, now considered a function of position *y* on the cell surface S, $\widehat {n} ( y ) $ is the unit vector normal to the cell surface at surface point *y*. We use the same singular perturbation method to get

38 $$ {r^{*}} ( y )\propto\frac{f ( x+y )r}{1+\kappa f ( x+y )}=\frac{f ( x )r}{1+\kappa f ( x )}+r{y^{T}} \nabla\frac{f ( x )}{1+\kappa f ( x )}+O \bigl( {{\Vert y \Vert }^{2}} \bigr) $$

where *x* is the position of the center of the cell, *κ* is the equilibrium association constant, and *f*(*x*) is the concentration of chemokine at *x*. The expression to the right of the equals sign results from a Taylor expansion.

Substituting Eq. (38) into Eq. (37) gives

39 $$ \varGamma=\chi\frac{r\nabla f ( x )}{{{ [ 1+\kappa f ( x ) ]}^{2}}} $$

where the constant *χ* is the chemotactic coefficient.

Finally, we let the coefficients *ζ*,*χ*, and *σ* become very large while their ratios remain constant. We further assume that there are no external forces. In this limit, we get the system of equations

40 $$ \begin{aligned}[c] X &=V\,dt \\ dV &=-\gamma V\,dt+\chi\frac{r\nabla f ( x )}{{{ [ 1+\kappa f ( x ) ]}^{2}}}\,dt+\sqrt{\gamma}\sigma \,dW \end{aligned} $$

where the parameters have been rescaled to give the form displayed.

Values for the motility parameters used in the simulation were calculated by fitting to data from 3D trajectory data of individual lymphocytes from 2-photon data (Kepler and Chan 2007).
