# Supplementary material for: A Model for Migratory B Cell Oscillations from Receptor Down-Regulation Induced by External Chemokine Fields
Source: Bull Math Biol. 2013 Jan 8;75(1):185–205. doi: 10.1007/s11538-012-9799-9 (PMC3547247; doi:10.1007/s11538-012-9799-9)
Supplement: Supplementary file 1 — Supplementary figures (PDF 166 kB) [file 11538_2012_9799_MOESM1_ESM.pdf]

# A Supplementary figures

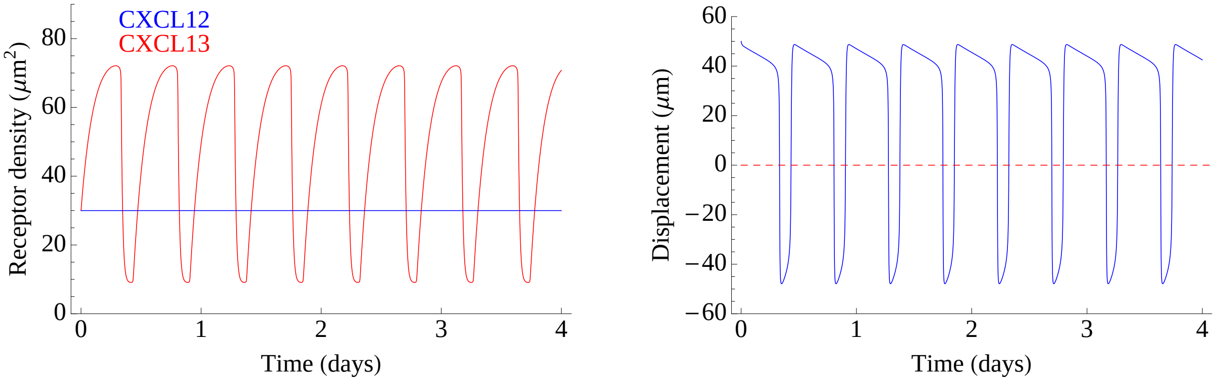


Figure A.1: Oscillations can occur in the germinal center model even when one receptor is fixed with $dr_2/dt = 0$. Parameters: $c = 10, \sigma = 26.5, \pi_1 = 0.5, \pi_2 = 0.1, \tau_1 = 0.06, \tau_2 = 0.06, \delta_1 = 0.006, \delta_2 = 0.006, \kappa_1 = 0.5, \kappa_2 = 0.1, \epsilon_1 = 0.3, \epsilon_2 = 0.3, \chi = 28, \gamma = 5, k = 50$.
